# Genetic Testing and Imaging in Men with Familial History or Genetic Predisposition of Prostate Cancer—Introducing the Prospective “ProFam-Risk” Study

**DOI:** 10.1016/j.euros.2025.12.018

**Published:** 2026-01-07

**Authors:** Jale Lakes, Matthias Boschheidgen, Gerald Antoch, Maike K. Klett, André Karger, Regina Roth, Silke Redler, Mangalore G. Pai, Dagmar Wieczorek, Bernadette Jäger, Tanja N. Fehm, Günter Niegisch, Tilman T. Rau, Peter Albers

**Affiliations:** a Department of Urology, Medical Faculty and University Hospital Düsseldorf, Heinrich Heine University Düsseldorf, Düsseldorf, Germany; b Centre for Integrated Oncology (CIO) Düsseldorf, CIO Aachen-Bonn-Cologne-Düsseldorf (ABCD), Düsseldorf, Germany; c Department of Diagnostic and Interventional Radiology, Medical Faculty and University Hospital Düsseldorf, Heinrich Heine University Düsseldorf, Düsseldorf, Germany; d Clinical Institute of Psychosomatic Medicine and Psychotherapy, Medical Faculty and University Hospital Düsseldorf, Heinrich Heine University Düsseldorf, Düsseldorf, Germany; e Institute of Human Genetics, Medical Faculty and University Hospital Düsseldorf, Heinrich Heine University Düsseldorf, Düsseldorf, Germany; f Center for Hereditary Breast and Ovarian Cancer, Medical Faculty and University Hospital Düsseldorf, Heinrich Heine University Düsseldorf, Düsseldorf, Germany; g Institute of Pathology, Medical Faculty and University Hospital Düsseldorf, Heinrich Heine University Düsseldorf, Düsseldorf, Germany; h Division of Personalized Early Detection of Prostate Cancer, German Cancer Research Center (DKFZ), Heidelberg, Germany

**Keywords:** Genetic risk, Multiparametric magnetic resonance imaging, Pathogenic germline variants, Personalized screening, Prostate cancer

## Abstract

Familial and genetic factors influence prostate cancer (PCa) risk, necessitating personalized prevention strategies. This study aims to establish and validate a prevention clinic (ProFam-Risk) for men with a familial or genetic risk of PCa, focusing on genetic testing, multiparametric magnetic resonance imaging (mpMRI), and psychosocial assessment. ProFam-Risk is a prospective registry and outpatient clinic at the University Hospital Düsseldorf, recruiting participants into three groups: healthy men with a familial risk (having two or more affected relatives or early-onset PCa), men with pathogenic germline variants (eg, *BRCA1/2*), and PCa-affected men meeting familial/genetic criteria. Participants undergo prostate-specific antigen testing, mpMRI, genetic analysis, psychosocial assessment, and receive risk-adapted recommendations for cancer prevention. Outcome measures include prevalence of pathogenic variants, PCa detection rates, and the impact of genetic counseling and mpMRI on clinical decision-making.

## Take Home Message

The ProFam-Risk outpatient clinic welcomes men with familial and genetic risks for prostate cancer. By combining prostate-specific antigen, multiparametric magnetic resonance imaging, and genetic analysis and psychosocial assessment, the clinic aims to personalize prostate cancer prevention and provide risk-adapted recommendations for this high-risk population.

## 1 Background

### 1.1 Introduction

Prostate cancer (PCa) is the most prevalent cancer among men in Germany and ranks as the second leading cause of cancer-related mortality [1]. Differentiating between aggressive and indolent forms of PCa is crucial, as early treatment of aggressive cases can lower the mortality and morbidity rates significantly. Various methods, including Gleason score, disease volume, prostate-specific antigen (PSA) density, and PSA velocity, have been employed to distinguish between these forms; yet, further refinement is necessary for personalized screening and treatment strategies. Certain demographics, such as men of African descent and those with a family history of PCa or specific pathogenic germline variants (eg, *BRCA1* and *BRCA2*), are at a higher risk of aggressive disease [2]. While genetic risk involves identifiable genetic germline variants, familial cases reflect a positive family history without specific pathogenic germline variants. The newly established prevention clinic ProFam-Risk focuses on the impact of pathogenic germline variants and family history in the context of PCa and their importance as genetic biomarkers in the era of personalized screening.

### 1.2 Role of family history and genetic risk in PCa

PCa has one of the highest hereditary components, with up to 58% of its risk attributed to genetic factors [3]. A family history of PCa is associated with a 2.5-fold increased likelihood of diagnosis and a two- to four-fold increased risk of mortality [4], with the risk escalating based on the number of affected relatives and their age at diagnosis [5]. Additionally, a family history of other cancers, particularly breast and ovarian cancer or colorectal cancer, correlates with an increased risk of clinically significant PCa [6], [7], [8]. Notably, 25% of men diagnosed with PCa report a family history of PCa or breast cancer [9]. Familial and hereditary PCa should be treated as two distinct clinical entities. While the hereditary form can be traced back to identifiable genetic alterations, the familial form has a positive family history but no identifiable genetic alterations. Therefore, ProFam-Risk seeks to address the critical need to incorporate genetic risk into prevention strategies.

### 1.3 Pathogenic germline variants

Nicolosi et al [10] reported a 17.2% prevalence of germline genetic variants in men with PCa, with *BRCA1/2* variants comprising 30% and *HOXB13* variants 4.5%. The prevalence of germline variants strongly depends on the individual cancer history of the patient. Pathogenic germline variants in BRCA2 or HOXB13 are associated with a two- to ten-fold increased lifetime risk of PCa [11], [12]. Other DNA repair genes, such as *CHEK2*, *ATM*, and *TP53,* and mismatch repair genes linked to Lynch syndrome (*MLH1*, *MSH2*, *MSH6*, *PMS2*, and *EPCAM*), are also associated with an elevated PCa risk, early-onset disease, and aggressive phenotypes [13], [14], [15], [16], [17]. *BRCA1/2* carriers face higher risks of metastasis and recurrence after radiotherapy, but not after radical prostatectomy, influencing the choice of local therapy [18], [19]. Additionally, mutation status impacts decisions on active surveillance, with *BRCA1/2* and *ATM* carriers more likely to experience disease upgrading during surveillance [20]. This underscores the importance of integrating genetic biomarkers not only in screening, but also in treatment planning.

### 1.4 Impact beyond PCa

The psychological implications of a familial or genetic risk are noteworthy, as men with a family history of PCa often overestimate their risk and express a strong interest in genetic testing [21]. However, being a mutation carrier can also lead to increased levels of distress [22].

Furthermore, germline testing for PCa also includes testing for genes associated with hereditary syndromes, such as hereditary breast and ovarian cancer (HBOC) or Lynch syndrome. Therefore, an interdisciplinary approach including comprehensive genetic counseling is mandatory.

### 1.5 Germline testing in PCa

The criteria for genetic testing in PCa differ based on guidelines and expert consensus, such as those from the Philadelphia Prostate Cancer Consensus Conference and the National Comprehensive Cancer Network (NCCN) [23], [24], [25].

The testing criteria consider personal medical history and family background, particularly the number and age of affected relatives. The specific genes recommended for testing can vary. The NCCN guidelines recommend testing for genes, including *BRCA1, BRCA2, ATM, CHEK2, PALB2, HOXB13, MLH1, MSH2, MSH6*, and *PMS2*, for eligible men. Nicolosi et al [10] evaluated the NCCN guidelines, finding that among 3607 men with a personal history of PCa, 620 (17.2%) had pathogenic variants, with 229 (37.0%) not meeting the NCCN testing criteria. Thus, the question of who should be tested remains unresolved.

### 1.6 Screening in carriers of genetic variants

The next critical question is what screening options are available for men with known pathogenic germline variants. Most professional organizations recommend shared decision-making with health care providers to create a personalized PCa screening plan. The NCCN guidelines advise annual PSA screening for *BRCA1/2* and *HOXB13* carriers starting at the age of 40 yr [24].

The Identification of Men with a Genetic Predisposition to Prostate Cancer (IMPACT) study has focused on PSA screening for *BRCA1/2* carriers. This prospective study recommended biopsies for men with PSA levels above 3.0 ng/ml. Interim results indicated a higher cancer incidence in *BRCA2* carriers than in noncarriers, but no imaging techniques, such as multiparametric magnetic resonance imaging (mpMRI), were integrated into this study [26].

Amini et al [27] evaluated a magnetic resonance imaging (MRI)-based screening strategy for pathogenic variant carriers using PSA, digital rectal examination, and mpMRI. MRI screening alone showed a higher net benefit than PSA screening alone, achieving a higher detection rate while reducing unnecessary biopsies. This highlights the crucial role of MRI in screening for pathogenic variant carriers.

## 2 Design and protocol

### 2.1 PCa prevention clinic for men at risk of familial PCa (ProFam-Risk)—study overview

In March 2023, the PCa prevention clinic ProFam-Risk started at the University Hospital Düsseldorf, marking Germany’s first clinic of its kind. ProFam-Risk serves as a prospective clinical registry to identify and validate genetic risk factors, while developing management recommendations for men at risk of PCa. The clinic provides specialized diagnostics, including family history evaluation, PSA testing, mpMRI, psychosocial assessments, and genetic analysis, to assess each patient’s risk. Its goal is to offer individualized risk assessment, screening, and treatment recommendations for men with a familial or genetic PCa risk. This initiative is a collaborative effort involving the Department of Urology, Institute of Human Genetics, Department of Diagnostic and Interventional Radiology, and the Clinical Institute of Psychosomatic Medicine and Psychotherapy at the University Hospital Düsseldorf. ProFam-Risk (NCT05681416) was approved by the local ethics committee (no. 2022-2051).

### 2.2 Aims and objectives

First, we aim to establish and validate a personalized counseling and monitoring strategy for men at an increased risk of PCa due to a positive family history or pathogenic genetic variants.

This includes assessment of psychosocial factors as part of the psychosocial subproject ProFam-Psych (a risk-adapted prevention clinic for familial and genetic PCa: psychosocial effects), funded by the German Cancer Aid [28]. ProFam-Psych aims to evaluate the longitudinal psychosocial trajectories associated with this PCa prevention clinic. Second, we seek to identify either likely pathogenic or pathogenic variants in known cancer susceptibility genes or novel cancer signaling pathways by discovering previously unreported PCa genes through extensive sequencing of PCa patients and family members. Third, we plan to evaluate mpMRI for PCa prevention in high-risk cohorts, similar to its role in women at a familial risk of breast cancer. This knowledge will enhance patient care and inform early detection and genetic counseling.

### 2.3 Eligibility and inclusion criteria

Eligible men are prospectively recruited in one of the three groups of the registry and are invited to the outpatient clinic in the Department of Urology. Group 1 includes PCa-unaffected men over 40 yr of age with an increased familial risk of PCa, defined by at least two first-degree relatives with PCa at any age or one first-degree relative with early-onset disease. Group 2 comprises PCa-unaffected men over 18 yr of age with a confirmed pathogenic germline variant associated with PCa, including *BRCA1/2* or other PCa-related genes. Group 3 consists of men over 40 yr of age with diagnosed PCa and a familial and/or genetic risk based on family history and/or confirmed pathogenic germline variants (Table 1).

**Table 1:** Inclusion criteria

Group 1	PCa-unaffected men aged >40 yr with a familial risk of PCa: 1. Two or more first-degree relatives with PCa diagnosed at any age 2. One first-degree relative with early-onset PCa (before age 60 yr)
Group 2	PCa-unaffected men aged >18 yr with a genetic risk of PCa: 1. Confirmed *BRCA1* or *BRCA2* germline pathogenic variant 2. Other confirmed PCa-associated germline pathogenic variants (*ATM*, *HOXB13*, *EPCAM*, *MLH1*, *MSH2*, *MSH6*, *PMS2*, *TP53*, *NBN*, *CHEK2*, *ATR*, *BRIP1*, *FANCA*, *GEN1*, *PALB2*, *PTEN*, *RAD51C*, and *RAD51*)
Group 3	PCa-affected men aged >40 yr with a familial and/or genetic risk: 1. Familial risk: one first-degree relative with PCa diagnosed at any age 2. Genetic risk according to the criteria in group 2

PCa = prostate cancer.

### 2.4 Recruitment/announcement

The project began with the announcement through various communication channels. A new website has been established to provide information about the study. Men are informed via flyers distributed to urologists, information letters, and collaboration with patient advocacy groups. The Düsseldorf branch of the HBOC consortium identifies men with likely pathogenic or pathogenic variants in *BRCA1 or BRCA2,* who are then contacted about the clinic. Additionally, men with known pathogenic variants in other known cancer susceptibility genes, for example, *MLH1, MSH2, MSH6*, and *PMS2*, are invited by the Department of Human Genetics. The Urology Department at the University Hospital Düsseldorf diagnoses and treats a large number of PCa patients on a yearly basis. From this collective, patients with a positive familial history of PCa or a known pathogenic variant are invited to participate in group 3 of the registry.

### 2.5 Study program

During the first on-site study visit, participants rotate through three departments at the University Hospital Düsseldorf. First, they are seen at the urology department, where informed consent is obtained. The participating men are thoroughly counseled about the consequences of genetic testing as well as of intensified screening, including the risk of overdiagnosis, prior to all procedures. Participants first complete the psychosocial assessment (eg, PCa-specific anxiety) using validated questionnaires, followed by questionnaires on medical history and pedigree information. Next, a clinical investigation is performed, made up of, for example, a physical examination; transabdominal ultrasound of the kidneys, bladder, and prostate; and transrectal ultrasound of the prostate. Blood samples are collected for PSA measurement, genetic analysis, and biobanking for future research.

**Fig. 1:**
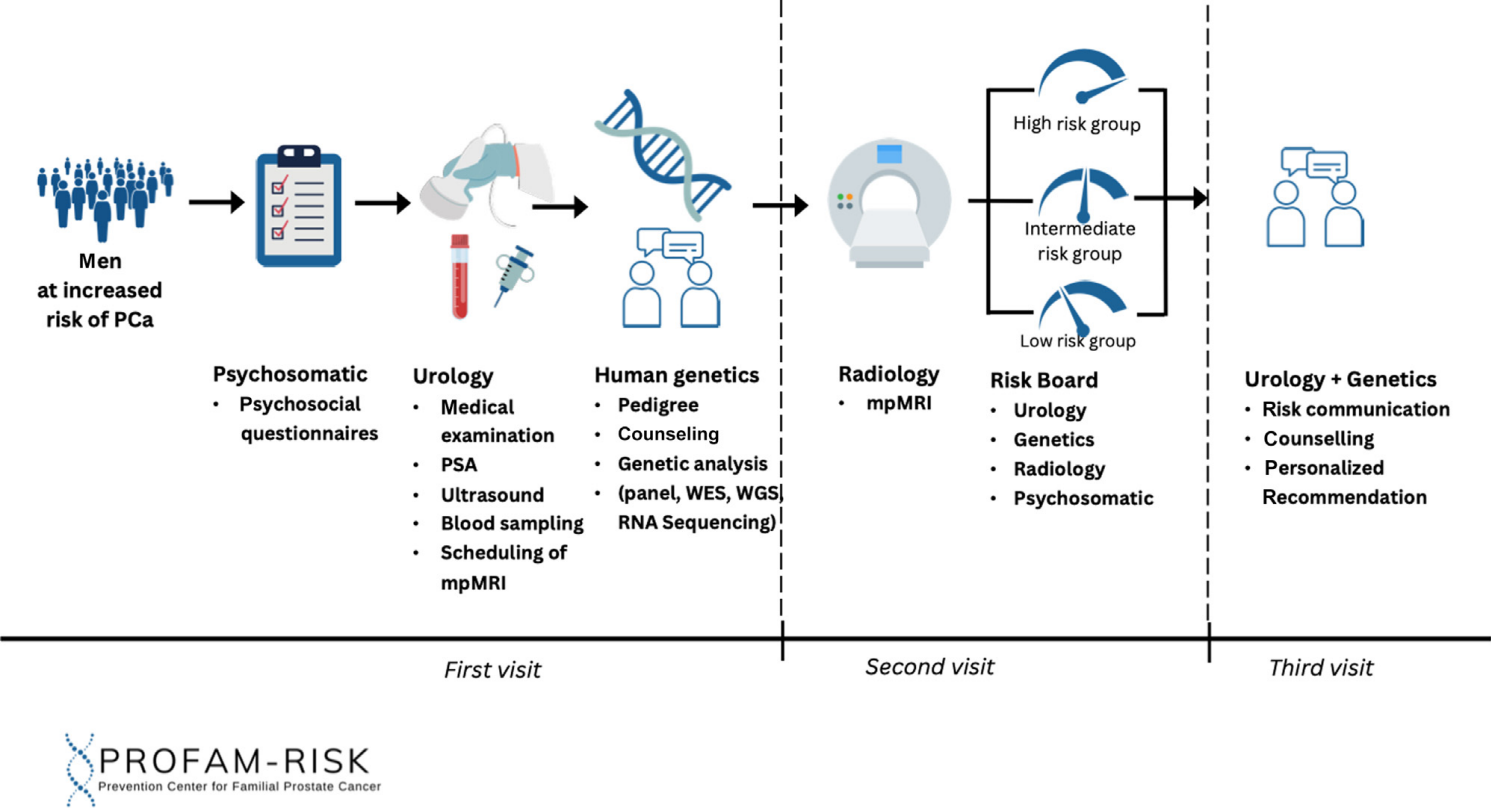
Study program. mpMRI = multiparametric magnetic resonance imaging; PCa = prostate cancer; PSA = prostate-specific antigen; WES = whole exome sequencing; WGS = whole genome sequencing.

Participants then receive genetic counseling in the Institute of Human Genetics; those in group 2 will not undergo genetic profiling but can request counseling. Finally, all participants without a history of PCa (groups 1 and 2) undergo mpMRI of the prostate, regardless of PSA levels. The mpMRI will include contrast-enhanced imaging, and prostate lesions are scored using the Prostate Imaging Reporting and Data System (PI-RADS) v2.1 [29]. The quality of imaging and reporting is evaluated using the Prostate Imaging Quality (PI-QUAL) criteria at the Department of Diagnostic and Interventional Radiology at the University Hospital Düsseldorf (Fig. 1).

### 2.6 Genetic testing

Men in groups 1 and 3 are offered germline testing to assess their genetic risk for PCa. This testing occurs in a diagnostic setting according to the German gene diagnostic law, through a three-step process, preceded by extensive genetic counseling. Advantages and disadvantages of genetic testing are explained to the participants before undergoing the test to ensure informed consent because of the potential psychological impact of the results as well as possible implications for their family members. In addition, they should be aware of the possible consequences for insurance, employment, and medical decision-making.

First, next-generation sequencing is used for a multigene panel analysis. In group 1, healthy individuals seeking counseling are tested, while in group 3, registry participants undergo primary testing. The analysis includes genes with confirmed associations with PCa, comprising a targeted panel of 20 established cancer susceptibility genes (*ATM*, *BRCA1*, *BRCA2*, *EPCAM*, *HOXB13*, *MLH1*, *MSH2*, *MSH6*, *PMS2*, *TP53*, *NBN*, *CHEK2*, *ATR*, *BRIP1*, *FANCA*, *GEN1*, *PALB2*, *PTEN*, *RAD51C*, and *RAD51D*).

We estimate that 80–85% of healthy men at risk of PCa will not have any pathogenic variants identified by the 20-gene panel. Only these participants proceed to whole exome sequencing (WES). Literature suggests an additional 10% to harbor pathogenic variants in other genes. For the remaining 70% of participants without findings from the panel or WES, whole genome sequencing (WGS) and a transcriptome analysis are conducted to identify novel genomic alterations related to PCa. Progress in genomic medicine has led to the identification of a large number of genetic variants the pathogenicity of which is unclear. These are termed “variants of uncertain significance” (VUSs) according to the American College of Medical Genetics and Genomics criteria, and constitute a dilemma for both patients and their relatives. In tumor entities for which the genetic basis is understood only rudimentarily, the need to perform advanced genetic analyses is particularly compelling when a VUS is detected. Under the German Genetic Diagnostics Act, men must provide written consent regarding disclosure of incidental findings and VUSs beyond the test panel; if consent is given, such findings are reported, including pathogenic variants in tumor risk genes and VUSs.

WES, WGS, and transcriptome library preparation are performed at the West German Genome Center in Cologne or Düsseldorf, funded by the German Research Foundation.

### 2.7 Risk stratification and management groups 1 and 2

**Fig. 2:**
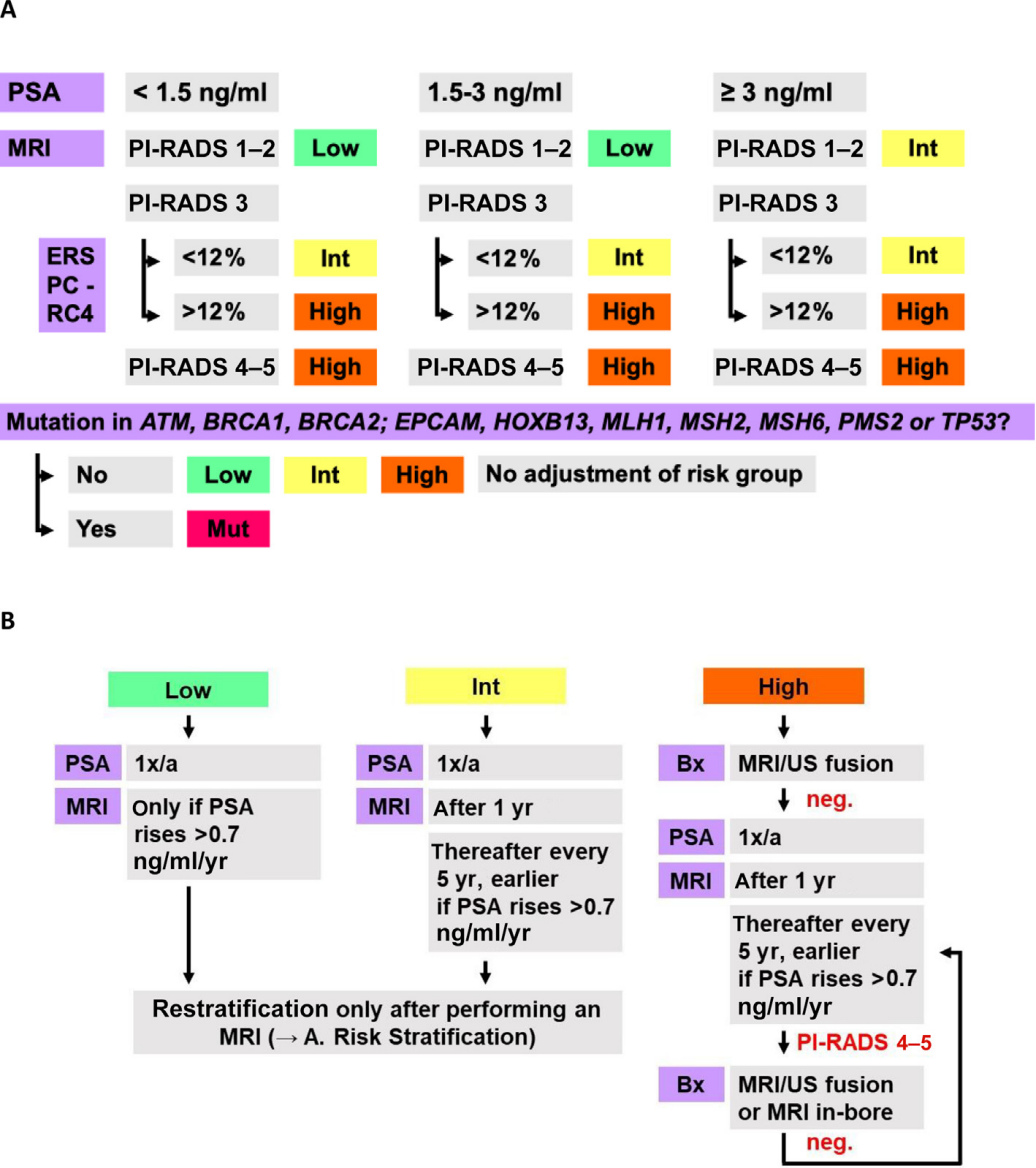

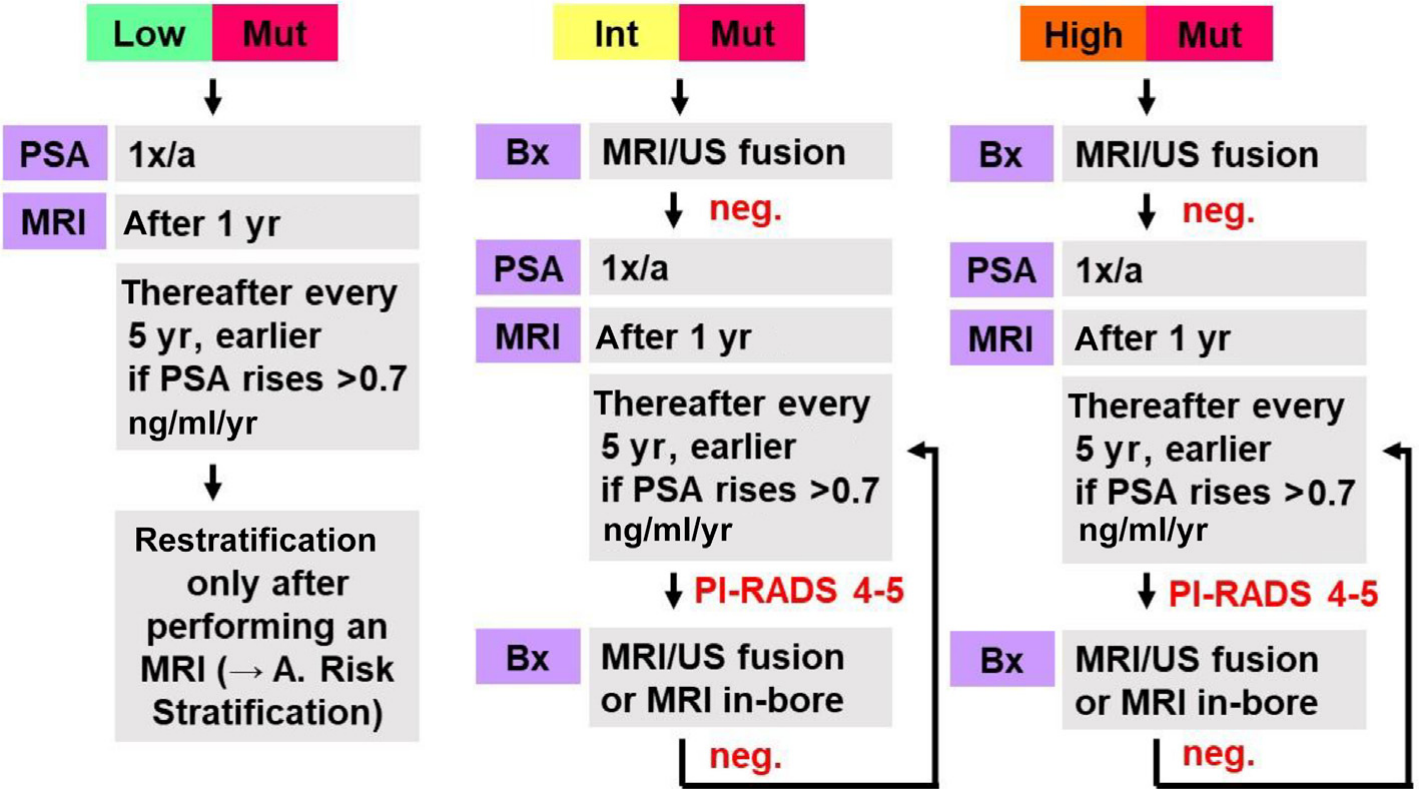
(A) Risk stratification in groups 1 and 2. (B) Follow-up management in groups 1 and 2. Bx = biopsy; ERSPC-RC4 = European Randomized Study of Screening for Prostate Cancer risk calculator 4; High = high risk; Int = intermediate risk; Low = low risk; MRI = magnetic resonance imaging; Mut = mutational burden; neg. = negative; PI-RADS = Prostate Imaging Reporting and Data System (version 2.1); PSA = prostate-specific antigen; US = ultrasound; 1x/a = once per year.

Once all clinical, genetic, and imaging results are available, individuals are reviewed in the “risk board”—an interdisciplinary conference involving human geneticists, urologists, radiologists, and psychologists to provide tailored recommendations for each man. Based on the published data from large population-based screening trials, all men in groups 1 and 2 are stratified in risk groups in a stepwise manner according to their PSA levels at presentation, mpMRI results, and European Randomized Study of Screening for Prostate Cancer risk calculator 4 results [30], [31], [32]. Additionally, participants are categorized by the presence of known or newly diagnosed variants (Fig. 2A).

Based on the risk group and mutation status, participants receive recommendations for PSA or MRI follow-up, or a prostate biopsy, if necessary. In case a biopsy is required by the study protocol, it is conducted as an MRI-ultrasound fusion biopsy at the Department of Urology. In-bore MRI biopsies are offered to men with suspicious MRI findings but a negative biopsy at the Department of Diagnostic and Interventional Radiology. After defining the initial stratification and personalized monitoring strategy, participants are seen at least annually, receiving a PSA test. Participants of group 2 aged under 40 yr are seen every 2 yr only up to the age of 40 yr. Follow-up MRI scans are prompted by initial risk classification or rising PSA levels, and unlike the initial MRI, are performed as biparametric MRI to minimize repeated application of intravenous contrast agents. Contrast material is added only if suspicious findings are observed during the scan, to minimize repeated application of intravenous gadolinium-based contrast agents (Fig. 2B).

Counseling of participants diagnosed with PCa within the ProFam-Risk clinic is based on the results of genetic testing. Men without pathogenic variants receive the standard of care. Men with pathogenic variants are counseled on local treatment options. With regard to active surveillance, participants are informed about the increased risk of disease upgrading, and the decision for active surveillance is evaluated more critically than in men without genetic alterations. All treatments and follow-ups are provided through the Department of Urology at the University Hospital Düsseldorf.

### 2.8 Management group 3

Group 3 of the prospective registry includes men with histologically confirmed PCa and a positive family history and/or a genetic predisposition to PCa. Most are expected to be already treated or currently under treatment. The aims of this group are to identify PCa susceptibility genes in affected patients and to offer extended screening to their family members within the PCa prevention center and the registry framework.

Counseling of group 3 participants is based on the results of genetic testing. Men without pathogenic variants receive the standard of care or standard follow-up. Men with pathogenic variants after local treatment receive standard follow-up and are informed about potential future targeted therapies such as PARP inhibitors. Men with pathogenic variants under active surveillance are counseled about the increased risk of disease upgrading and the need for critically re-evaluating continuation of active surveillance. All treatment is provided through the Department of Urology at the University Hospital Düsseldorf.

### 2.9 Biobank

To enable translational research projects within this very relevant subpopulation at a familial or genetic risk of PCa, liquid collection (blood and urine) alongside the clinical registry was established. As part of this prospective registry, a tissue collection biobank of the participants will be built. In cases of tissue processing, extractions from formaldehyde-fixed paraffin-embedded tissue or fresh frozen tissues will be registered simultaneously.

### 2.10 Statistical analysis

The ProFam-Risk study is designed as a prospective clinical registry and does not include predefined endpoints. Accordingly, the statistical analysis will primarily be descriptive. Continuous variables (eg, age, PSA levels, and prostate volume) will be summarized by mean, median, standard deviation, and interquartile range. Categorical variables (eg, family history, germline variants, and MRI findings) will be reported as absolute numbers and percentages. No hypothesis-driven statistical testing is planned at this stage, as the primary aim of the registry is to generate real-world data and describe the spectrum of clinical, genetic, imaging, and psychosocial findings in men at an increased familial or genetic risk of PCa. This descriptive approach is essential for providing an overview of patient characteristics and outcomes, and will serve as a foundation for future hypothesis-generating analyses and interventional trials.

### 2.11 Outreach

In addition to the Düsseldorf prevention clinic, the ProFam-Risk team employs outreach strategies to enhance study enrollment across Germany. Besides cooperation with national patient advocacy groups and online services, ProFam-Risk is involved in the ONCONNECT outreach project by German Cancer Aid, which aims to provide local support and points of contact at other comprehensive cancer centers and establish similar prevention clinics modeled after ProFam-Risk.

### 2.12 Current accrual in ProFam-Risk

By July 2025, a total of 123 men had attended the prevention clinic. Among them, 25 had a family history of PCa, qualifying them for group 1, while 32 participants had known pathogenic germline variants (13 with *BRCA2*, 12 with *BRCA1*, one with *MLH1*, one with *CHEK2*, one with *MSH2*, one with *MSH6*, and three with *ATM*). Additionally, 66 men with a known PCa and familial risk (group 3) visited the clinic, 22 of whom were under active surveillance at the time. The age and PSA distributions are shown in Table 2.

**Table 2:** Age and PSA distribution of all participants

	All	Group 1	Group 2	Group 3
*n*	123	25	32	66
Mean age at consent (range)	59 (19–79)	53 (41–83)	48 (19–74)	61(45–79)
Mean age at diagnosis (range)				59 (45–74)
PSA at consent (ng/ml; range)		1.61 (0.40–14.6)	0.93 (0.43–3.6)	
PSA at diagnosis (ng/ml; range)				5.6 (1.4–109.0)

PSA = prostate-specific antigen.

## 3 Discussion

The ProFam-Risk project represents an innovative step toward precision medicine in PCa prevention, targeting men with familial or genetic predisposition. By integrating genetic testing, mpMRI, and psychosocial assessment, the clinic addresses both clinical and psychological aspects of cancer risk. Despite promising first recruitment results, challenges such as limited sample size and cost effectiveness remain, warranting further research. The inclusion of other study locations is therefore essential.

### 3.1 Benefits

A major strength of the project lies in its interdisciplinary approach. The combination of genetic testing and mpMRI enables risk-adapted screening with the potential to improve early detection of clinically significant PCa, while simultaneously reducing unnecessary biopsies. Furthermore, the inclusion of psychosocial assessment ensures that the psychological burden of genetic risk, which often results in increased anxiety and uncertainty, is addressed systematically. The project also establishes a biobank, laying the foundation for future translational research that may identify novel biomarkers and therapeutic targets. In ProFam-Risk, all patients with *BRCA* variants were derived from the Düsseldorf branch of the HBOC consortium or the German HNPCC (hereditary nonpolyposis colorectal cancer) consortium, highlighting the need for closer collaboration to support male pathogenic variant carriers identified through female or colorectal cancer screening.

High-risk screening programs such as ProFam-Risk are complementary to population-based screening strategies rather than competitive alternatives. High-risk screening focuses on a biologically and clinically enriched subgroup defined by familial and/or genetic predisposition, in whom the probability of clinically significant disease is substantially higher. By integrating germline genetics, mpMRI, and individualized risk stratification, high-risk programs allow for more precise selection of candidates for intensified surveillance and invasive diagnostics.

### 3.2 Risks and limitations

Despite its promise, several challenges must be acknowledged. First, the relatively small sample size and single-center design may limit the generalizability of early findings. This limits the statistical reliability of PCa incidence estimates. Even in BRCA2 carriers, large cohorts are required, as demonstrated by the 5.2% PCa incidence reported in the IMPACT study [26]. Multicenter expansion within the ONCONNECT outreach project by German Cancer Aid will therefore be essential. The interpretation of mpMRI may be challenging in this younger prevention population [33]. Diffuse signal changes on T2- and diffusion-weighted imaging as well as diffuse and widespread contrast enhancement result in a larger number of PI-RADS 3 readings, potentially leading to unnecessary follow-up procedures [34]. One task to be addressed in ProFam-Risk will therefore be to define a workflow for follow-up in these men.

Furthermore, the identification of germline variants may generate psychological distress in carriers and place additional demands on genetic counseling resources. From a methodological perspective, the absence of a clearly defined endpoint and the need for long-term follow-up pose challenges for clinical validation.

### 3.3 Financial considerations

The financial sustainability of such a comprehensive prevention clinic is a central concern. Genetic testing, mpMRI, and interdisciplinary care are resource intensive and not yet reimbursed for asymptomatic high-risk individuals in most health care systems. The implementation of this model beyond the academic setting may be limited. Current support from the German Cancer Aid allows for pilot implementation, but future expansion to other centers and long-term follow-up will require substantial investment. Demonstrating cost effectiveness—through reduced overtreatment, fewer unnecessary biopsies, and earlier detection of aggressive cancers—will therefore be critical to justify broader adoption within public health care systems.

## 4 Conclusions

In summary, ProFam-Risk provides an important proof of concept for individualized PCa prevention in men with familial or genetic predisposition. While the benefits of integrating genetic, imaging, and psychosocial strategies are evident, the project faces future challenges. Long-term evaluation and multicenter expansion will be essential to confirm its value and secure sustainable health care integration. However, long-term validation of its model could enhance global screening strategies, reducing mortality of and improving care for high-risk individuals.

Additionally, ProFam-Risk may serve as a proof-of-concept project by demonstrating the feasibility, clinical utility, and added value of an integrated, interdisciplinary high-risk screening approach, including patient acceptance, psychological outcomes, and adherence to follow-up recommendations.

  ***Author contributions:*** Jale Lakes had full access to all the data in the study and takes responsibility for the integrity of the data and the accuracy of the data analysis.

  *Study concept and design*: Albers, Wieczorek, Karger, Antoch, Lakes.

*Acquisition of data*: Klett, Boschheidgen, Jäger, Fehm, Roth, Pai, Lakes.

*Analysis and interpretation of data*: Albers, Boschheidgen, Klett, Pai, Niegisch, Lakes.

*Drafting of the manuscript*: Lakes.

*Critical revision of the manuscript for important intellectual content*: Albers, Wieczorek, Karger, Antoch, Lakes, Boschheidgen, Klett, Redler, Niegisch, Rau.

*Statistical analysis*: Lakes.

*Obtaining funding*: Albers, Wieczorek, Karger, Antoch.

*Administrative, technical, or material support*: Pai, Boschheidgen, Klett, Roth, Jäger, Lakes, Rau.

*Supervision*: Albers, Antoch, Wieczorek, Karger, Fehm, Niegisch, Rau.

*Other*: None.

  ***Financial disclosures:*** Jale Lakes certifies that all conflicts of interest, including specific financial interests and relationships and affiliations relevant to the subject matter or materials discussed in the manuscript (eg, employment/ affiliation, grants or funding, consultancies, honoraria, stock ownership or options, expert testimony, royalties, or patents filed, received, or pending), are the following: Günter Niegisch: honoraria—Roche Pharma AG, Medac, AstraZeneca, Astellas Pharma, Pfizer, BMS GmbH & Co KG, Eisai Germany, Janssen Oncology, and MSD; consulting or advisory role—Roche Pharma AG, Bristol Myers Squibb, Janssen Oncology, Pfizer, Merck, Astellas Pharma, AstraZeneca, and Medac; travel, accommodations, and expenses—Janssen Oncology, Roche, AstraZeneca, and Merck; and publication support—Pfizer, BMS, and Ferring. Maike Klett: funded by the German Cancer Aid, part of the Cancer Prevention Graduate school of the German Cancer Research Center.

  ***Funding/Support and role of the sponsor:*** This study is funded by the German Cancer Aid (70114733) and by the German Research Foundation to Peter Albers and Dagmar Wieczorek (AL 391/9-1 and WI 1440/14-1).

  ***Acknowledgements:*** The authors are grateful to Anna Henrike Rabe for the administrative support in handling the invitations and the public announcement, Maxim de Vrieze for his help with the figures, and Fritz Böge for his support in establishing a liquid biobank.

  ***Use of generative AI and AI-assisted technologies:*** During the preparation of this work the authors used ChatGPT in order to proof read and improve language and readability. After using this tool/service, the authors reviewed and edited the content as needed and take full responsibility for the content of the publication.
